# COVID-19 induced shocks and its implications for human capital development

**DOI:** 10.1186/s12939-024-02119-1

**Published:** 2024-02-16

**Authors:** Abiodun Olusola Omotayo, Adebayo Isaiah Ogunniyi

**Affiliations:** 1 North-West University, Mafikeng, South Africa; 2 University of Ibadan, Oyo State, Nigeria

**Keywords:** Coronavirus, Education outcomes, Economic recovery, Health outcomes, Social safety nets, Socio-economic impact, Nigeria

## Abstract

**Background:**

COVID-19 has delivered an enormous shock to the global economy, triggering the deepest recession in eight decades, almost three times as deep as the 2009 global recession. Of all the nations in Africa, Nigeria remains one of the nations with a huge and significant impact on the human capital.

**Methods:**

Hence, here we employed the recent nationally representative data from Nigeria - the COVID-19 National Longitudinal Phone Survey 2020-World Bank Living Standards Measurement Study Integrated Agriculture Survey (LSMS-ISA), a harmonized dataset to explore how the COVID-19 induced shocks affected households’ human capital development (using health and education outcomes).

**Results:**

The results indicate that the COVID-19 induced shocks impact on both health and education in Nigeria. Interestingly, access to social safety nets had a positive association with the health and education outcomes. The study concludes that households’ access to social safety nets, particularly during the COVID-19 pandemic aids in the development of the nation’s human capital. Therefore, effectively enhancing household’s resilience and strengthening human capital development require positive and considerable innovation, maybe over a period of years. Hence, just an access to the national social safety nets programs or social programs may not be as effective as expected. Therefore, it may not be as successful as intended to just have access to national social safety net programs or social programs that contribute or transfer negligible amounts to the vulnerable recipients over short time frames.

## Introduction

The world economy has experienced a tremendous set back as a result of COVID-19, causing the deepest global recession in eight decades that is almost three times the one that began in 2009 [1–3]. With well over four million fatalities and millions of people suffering from diminished opportunities and disrupted livelihoods, the pandemic’s toll on health, education, skills, and human life has only continued to rise [4–6]. As a result of the accompanying control measures’ significant compromise of the numerous factors necessary to generate an appropriate supply of accessible labor, health care, education, consumption, investment, and labor markets have all been severely curtailed [7–9]. Intriguingly, human capital remains one of the most inalienable assets an individual can hold [10–12].

Human capital is the fundamental asset through which individuals become productive member of a society and enables the society to thrive [13–15]. Sadly, the COVID-19 damaged this priceless asset. The COVID-19 pandemic left millions of people’s health poor, disrupted the education of hundreds of millions of children, exerting more negative effects on the developing economies than natural disasters, whose toll on physical capital vastly exceeds that on human capital [16–20]. Nigeria, one of the SSA countries, was not immune to the detrimental effects of the COVID-19, as the pandemic has stunted the nation’s economic growth potential due to its effects on the health and educational sectors, among other things.

Nigeria’s fundamental challenges (such as high poverty rate, poor educational and health qualities among others) are quite alarming. Due to this, given the COVID-19 outbreak, existing fragile progress made in education and health and other key SDGs -Ensuring healthy lives and wellbeing for all ages (SDG3); Quality Education (SDG4); decent work and economic growth (SDG8), were reversed. Hence, the 2030 agenda may be difficult to reach [21]. Thus, research that will direct investment in the health and education sector is crucial as adequate infrastructure can be put in place to device means for a successful health care and academic exercise in Nigeria. In this way, investment in education and health will help the Nigeria economy to return to normalcy.

We contribute to the existing body of literature by presenting a theoretical model and empirical findings that explore the impact of a sudden health-related shock on crucial economic development indicators, particularly education and health. While prior research has emphasized the overall impact of infectious diseases on the economy, it has often overlooked the sectoral implications of periodic infectious diseases. For example,Smith KM, Machalaba CC, Seifman R, Feferholtz Y and Karesh WB [22] evaluated the economic impact of infectious diseases within a multi-sectoral context, and [23], examined the socio-economic effects of emerging infectious diseases in Africa. Diverging from these studies, our focus is specifically on the sudden emergence of a pandemic and its economic impacts on specific human capital indicators, with a primary emphasis on education and health in the case of our study.

Given this development, the objective of this study is to broaden the understanding of the effect of the COVID-19, and offers a critical reflection that would enhance the human capital development in Nigeria. Additionally, the study tests the role of access to social safety nets on smoothening the effect of the pandemic on households’ human capital in Nigeria.

## Literature review

There is emerging evidence that the adverse effects of COVID-19 permeates the welfare of households and human capital development from different channels such as disruptions in access to school, loss of job or decrease in income, loss of sales from household business, and reduced availability for work due to reduced or lack of alternative care for children and sick household members [21, 24–29]. A joint statement by ILO, FAO, IFAD and WHO emphasized the massive loss of livelihoods as enterprises suffer existential crises and a significant proportion of the world risk losing their jobs due to the pandemic with the informal sector being more at risk as they have little to social protection, quality healthcare and productive assets [30–32].

With the rising poverty and setbacks on development outcomes, the COVI9-19 has been identified as a problem to the gains on human capital [33–35]. The rise in mortality rates associated with the pandemic has translated to the loss of primary care givers that has further made households susceptible to the threats of poverty, malnutrition, poor health, depression, violence, and child marriage [36, 37]. Children risk being among the biggest victims of the pandemic as the country specific orphanhood estimates by the Imperial College London revealed increasing rates of orphanhood across the globe due to COVID-19 [5, 38]. In Nigeria, as of 2021, about 4100 children in Nigeria have lost at least one primary giver, about 4000 have been orphaned while about 4700 have lost one or both parents, due to COVID-19 [37, 39, 40].

There is growing body of empirical studies that focused on the effect of COVID-19 on households’ livelihoods and its adverse effects on human capital investments. In assessing access to healthcare, studies such as [36, 41–44], identified that existing health inequalities have been further aggravated by the pandemic. The decline in income levels and increasing poverty among households has further strengthened the financial barrier of payments for health resulting in both unmet needs and financial hardship for people using health services [45–47]. Reduced access to health care has stems from strained health systems as the health sector remain underfunded globally [48] while the number of in-patients remains high due to the pandemic.

For education, many governments consider increasing access to education as a main strategy for Human capital development [49]. However, with the onset of COVID-19, many children have dropped out of school [50, 51], and many could not return to the classroom as their parents have lost their jobs due to COVID-19 [52]. This implies that with the rising poverty, more children were at increased risk of dropping out of school or being denied access all together. This is evident as several empirical studies [12, 53–55], that education expenditure increases with family income which has however been on the decline in many households due to the pandemic [5, 56, 57]. With the large number of students out of school, the pandemic reduced the possibility of achieving the SDG 4 -quality education [21, 58] and its far-reaching consequences may reverse the gains made in improving global education and the development of human capital [5, 21].

### Theoretical framework

The underlying framework of theories employed for the analyses of the research is the human capital development theory- education, health, and economic growth theory. In 1992, Mankiw, Romer, and Weil first augmented Solow RM [59] neoclassical growth model to incorporate human capital in education. Knowles S and Owen PD [60], further extended the neoclassical growth model by incorporating both health and education. Their results show a significant statistical relationship between health and growth with education having a modest role [61]. Additionally, McDonald S and Roberts J [62] supported the results found by Knowles S and Owen PD [60]. Sun et al. [63], affirmed technology and human capital as the key driving forces to promote economic growth. They proved also that higher workforce human capital led to a higher quantity of patents and a higher probability to innovate and therefore human health.

More recently, [61, 64], studied the effect of health and education on economic growth in MENA economies, and the results of his study showed that education has a positive and significant effect on economic growth at long run. However, health has a negative but negligible influence on such growth in MENA countries. As well, the findings reported by [65, 66], are different and indicated that growth-oriented policies should favor investments in education over health. Subsequent to this, [65–67], show that both health and education have positive significant effects on economic growth in China and East Asia.

### Intervention and the adverse impacts of COVID-19 shocks

The importance of developing human capacity and its ability to have a positive impact on national development requires governance that is characterized by proactive action, competence, pragmatism, negotiation, and political will [68]. According to Loss J [69], Intervention is regarded as a comprehensive approach encompassing various elements and activities aimed at achieving the desired outcomes of a programme. Loss contends that interventions employing multiple strategies are the most effective in bringing about the intended and long-lasting change, as they have the potential to reach a larger population through diverse means. By influencing individuals’ knowledge, attitudes, beliefs, and skills, as well as enhancing their social status, and establishing supportive environments, policies, and resources, interventions facilitate transformative change, according to Loss.

One interventionist approach is targeting strategy. Interventionist strategy is a tool of government in enhancing the closing of empowerment gaps or cushioning the impact of a shock such as the COVID-19 pandemic in the society. The identified gaps are closed by targeting mechanism, which is inherently restrictive. Every policy has targeting undercurrent [70]. For instance, economic, health and education policies target the poor and the illiterate during the COVID-19 pandemic in Nigeria. It is a focused group policy that delineates population needs. However, in this case targeting is a necessary as it enhances efficient management of limited resources arising from its goal-oriented character leading to access restriction to the non-target group.

Intervention is a people-oriented drive that requires all-inclusive approach, its broad application transcends numeric characteristic to include economic, educational, and health capabilities of the Nigerians during COVID-19 pandemic. It is only when these conditions are in place that well-intended intervention can occur. People-centered interventions for positive change proceed from participation of real target groups and implementing agencies that possess requisite skills, competencies, and capabilities in policy initiation, implementation, monitoring, and evaluation processes. Local populations are comprehensively engaged in the entire policy process. Leaders, however, have been shaped by the emergent focus on governance in ensuring efficiency in the management of resources such as the provision of social safety nets for public good. Importantly, involvement of empowered stakeholders and issue-focused approach such as the COVID-19 pandemic is rapidly succeeding representative participation [68, 71].

## Material and methods

### Study area

**Fig. 1:**
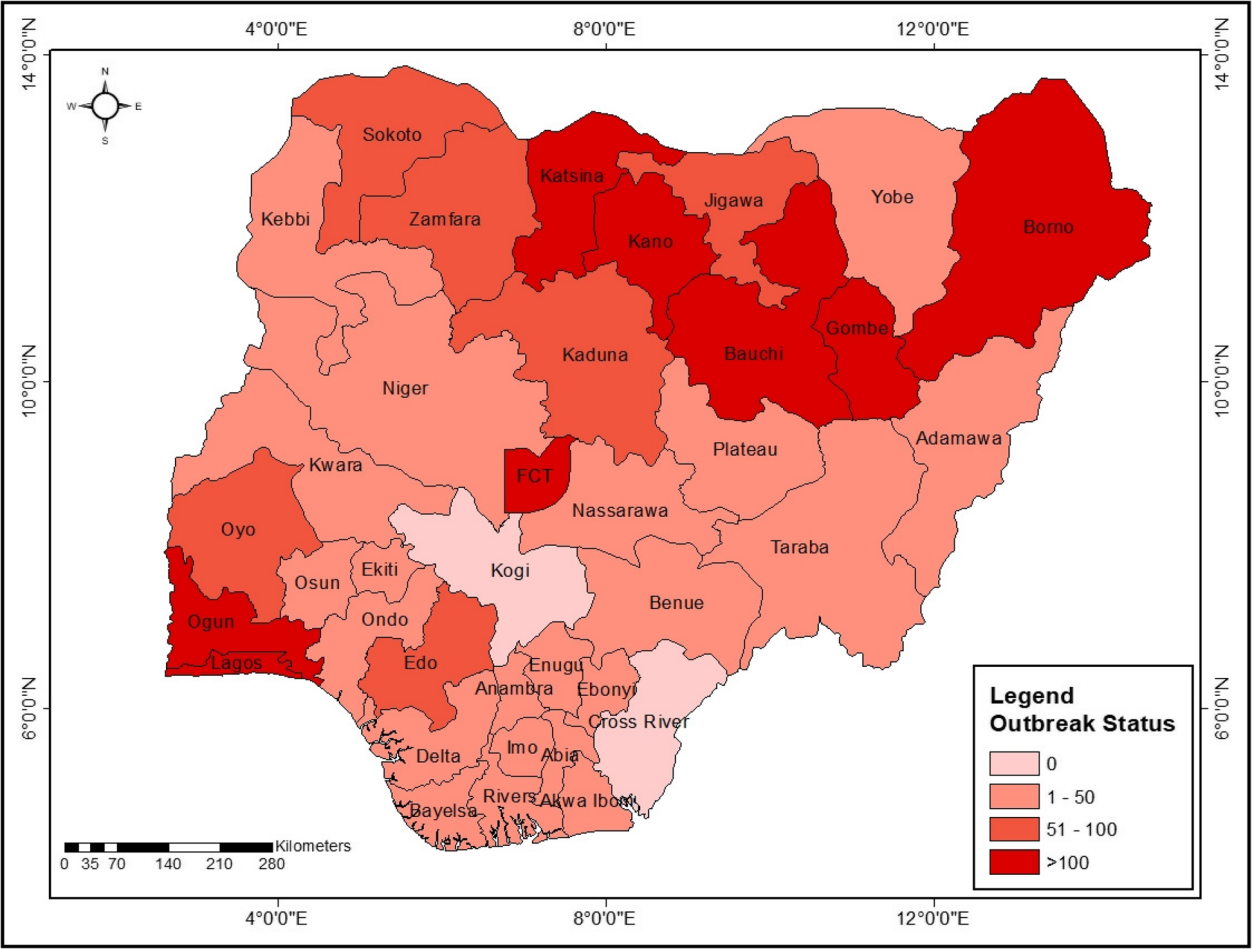
Geographical locations showing the 34 states affected by COVID-19 in Nigeria

Nigeria is a lower middle-income nation in West Africa with a southern boundary that is formed by the Atlantic Ocean’s coastline. Nigeria is a federation made up of 36 states plus Abuja (Fig. 1), which serves as the Federal Capital Territory (FCT) [72–74]. Despite the significance of oil exports, agriculture continues to be the backbone of the economy, employing 36.5% of all laborers and providing a substantial source of income for the majority of the population. More so, according to estimates from Nicholas & Patrick (2015), 52% of Nigeria’s population lives in rural areas, compared to 48% of urban people. Despite a recent economic downturn, the agriculture sector’s value added—21% of GDP—remains comparatively strong [73]. The nation touts having Africa’s greatest economy, with a US$479 billion projected GDP. With oil and mining excluded, GDP growth is predicted to increase by 6.1%, thanks to robust performances in professional services, business, and agriculture. The primary exports of the nation are crude oil, petroleum by-products, cocoa, and rubber.

According to estimates, oil and gas revenue has decreased by 14.4% since 2013. About 90% of Nigeria’s exports and 75% of its budgetary income come from oil. On February 27, 2020, Nigeria reported its first case of COVID-19. Since then, the illness’s prevalence and fatality rate have steadily increased [75, 76]. Since they provide care and are therefore susceptible to the disease, healthcare personnel were crucial in the fight against COVID-19 infection [75, 77]. Thus, in order to slow the spread of the pandemic, Nigeria, like other nations, implemented a partial or total lockdown [75]. Additionally, the pandemic shocks and associated economic disruptions on the already precarious progress made in education and health as well as other crucial SDGs 3, 4, and 8 in Nigeria have caused the previously obtained progress to be undone, which further impedes favorable outcomes for the development of human capital. Therefore, achieving the SDGs in Nigeria may be challenging under the 2030 Agenda [21].

### Data and descriptive statistics

Our primary data originated from the Nigerian Living Standards Measurement Study Integrated Agriculture Survey (LSMS-ISA), which was conducted by the World Bank and the Nigerian Bureau of Statistics. We used data from 4 rounds of longitudinal household surveys: 1 pre-COVID-19 in-person survey and 3 post-COVID-19 phone surveys (rounds 1, 3, and 8 of the surveys primarily take into account the shocks variable). The dataset is robust, representative and offer in-depth details on the characteristics, shocks, education, health, and livelihood outcomes of individuals and households. Pre-COVID-19 survey data were primarily gathered in January and February 2019, and post-COVID-19 phone survey data were primarily gathered between April and November, 2020. The 2019 LSMS-ISA survey’s post-COVID-19 survey sought to follow up on the households surveyed there. In the most recent interview round (2019), 4976 households made up the overall sample, and 99.2% of them gave phone numbers.

A random sample of 3000 homes was chosen for the phone survey from among those possessing phone numbers. About 69% of the households were successfully reached while 94% (1958) of these households were able to conduct successful interviews (Nigerian Bureau of Statistics and World Bank, 2020; World Bank, 2020). After data cleaning and mining, we were able to get 1725 households, which serves as our final sample. Data from the phone survey and the prior round were then combined to generate a household-level panel data set. A sum of 6900 were produced by our team. We present in Table 1, our outcome (health and education) and interest variables.

**Table 1:** Summary statistics of the outcome and interest variables (Pooled sample)

**Variables**	Mean	Standard deviation
**Outcome variables**		
***Health***		
Access to medical services during COVID-19, 0/1	0.32	0.20
Ability to pay for the medical, 0/1	0.20	0.19
***Education***		
Engagement in any education activities during COVID-19	0.63	0.11
Ability to pay for session lesson tutor for the children, 0/1	0.07	0.02
Ability to use mobile learning application, 0/1	0.20	0.01
Listen to educational programme on radio, 0/1	0.16	0.04
**Interest variables – Shocks induced by COVID-19**		
Shock count (number)	2.60	1.51
At least one shock	0.94	0.23
Job loss of household head	0.14	0.11
Theft/looting of cash and other property	0.10	0.30
Increase in price of farming/business inputs	0.64	0.22
Fall in the price of farming/business output	0.16	0.01
Disruption of livelihood activities	0.20	0.39
Increase in price of major food items consumed	0.89	0.31
Illness or death of income earning member of household	0.16	0.03
Observations	6840	

### Empirical strategy and model specification

In this section, we describe the empirical methodology and specification used to pinpoint the effects of COVID-19-induced shocks on households, with a focus on the consequences for the growth of household human capital. We were interested in both the immediate impact of COVID-19-induced shocks as well as how much it impacted the growth of household human capital. Studies on human capital distinguish between transient and permanent shocks [78–82]. These shocks’ theoretical impact and choices for how to respond vary. For instance, if we think that COVID-19-induced shocks (such losing a job or experiencing other financial difficulties) decrease children’s attendance at school and may lead to an increase in child labor, participation outcomes (with a stronger expectation among the poor), a permanent negative shock to increase child labor is expected, especially for children in poor households.

On the other hand, social safety nets can be a useful instrument for mending temporary shocks. Instead, due to the frequently irreversible effects of interruption in education, lowering a child’s attendance at school in reaction to a transient shock has significant negative effects on the development of human capital. In this study, we concentrated on temporary shocks, which were represented by two distinct variables. First, a dummy variable that captured whether a household had experienced at least one of the identified shocks in the data set was used to measure shocks. Second, we calculated the magnitude of the shocks and the variety of shocks that the households had to deal with (we go into more depth about these shocks’ features later).

The shocks that are specifically available in the data and used include job loss, theft/looting of money and other property, an increase in the price of farming/business inputs, an increase in the price of farming/business output, a disruption of livelihood activities, an increase in the price of major food items consumed, and illness, injury, or death of a household income earner. In addition to other control variables, we looked at how social safety net accessibility affected the result variables. In light of this, we anticipate that improved access to social safety nets will slightly mitigate the impact of transient shocks. The policy ramifications of this finding are important; increasing household access to social safety nets can help lessen the impact of shocks caused by COVID-19 and subsequently lower ineffectively high levels of poor human capital. We examined the effect of COVID-19 induced shocks on household human capital development. Our basic specification is:

1 $${HCD}_{ijt}= {\beta }_{0}+ {\beta }_{1} {X}_{ijt}+{\beta }_{2}{Covidshock}_{ijt}+ {\varepsilon }_{ijt}$$

Where the subscripts represents individuals $(i)$, living in households $(j)$, and survey rounds were represented by $(t=1,\dots ,T)$; ${HCD}_{ijt}$ is the human capital development indicators, ${Covidshock}_{ijt}$ is our measure of the COVID-19 induced shocks (discussed in detail in the next section), and ${X}_{ijt}$ contains a set of controlling variables which the individual, household, and community characteristics. We expect transitory shocks to lead to a decrease in human capital development especially if the social safety nets are limited or non-existent, i.e. we expect ${\beta }_{2}>$ zero.

By way of construction, we first estimated this equation using OLS, pooling all rounds of our panel survey and allowing for household-level clustering. There are numerous possible dimensions of selection along unobservable factors, even though this specification accounts for a large variety of observable individual and family traits and includes community dummies. Poor households, for instance, may be more susceptible to COVID-19-induced shocks because they are less equipped to deal with their effects and may lack the resources to do so, while also being more likely to send their children to work because, prior to COVID-19, many (especially in rural areas) may have been sending their children to formal education. To address the problem of selection on unobservable, we will allow for fixed effects and estimate the following:

2 $${HCD}_{ijt}= {\alpha }_{j}+ {\delta }_{t}+{\gamma }_{w}+ {\beta }_{1} {X}_{ijt}+{\beta }_{2}{Covidshock}_{ijt}+ {\varepsilon }_{ijt}$$

where ${\alpha }_{j}, {\delta }_{t}, {\gamma }_{w}$, represents the household fixed effects, time fixed effects, and a fixed effects survey. Therefore, within-household variation adjusting for time and survey round effects was used to estimate the model. Keep in mind that at the home level, fixed effects include unobservable qualities at the community level. We cannot, however, rule out time-varying unobservable household-level factors.

## Results and discussion

### Respondents’ socio-economic characteristics

We present in Table 2 the descriptive statistics of the respondents. The result shows that 15.35% of the respondents falls within the age category of between 18 and 35 years. This age range represents the African Union definition of youth in Africa. The result suggests that evidence from this study can be apply to both the youth and adult. More than 80% of the respondents were male and suggesting that most of the households are male-headed which is not a deviation from expectation that most households in Africa are headed by male [83–85].

**Table 2:** Description of the respondents

Variable	Description	Number	Percentage (%)
Age Range	18–35	1,060	15.36
	36–45	1,768	25.62
	46–55	1,585	22.97
	55–65	1,201	17.41
	Above 65	1,286	18.64
Average			
Gender	Female	1,146	16.75
	Male	5,694	83.25
Sector	Rural	60.89	4,199
	Urban	39.11	2,697
Marital Status	Married	4950	72.37
	Divorced	178	0.26
	Widowed	1134	16.58
	Single	738	10.79
Household Size	1–5	2069	30.26
	6–10	3941	57.63
	11–15	666	9.74
	Above 15	162	2.37
**Average = 6.47, SD = 3.79**			
Education	No Formal Education	1205	17.63
	Primary	2412	35.26
	Secondary	1566	22.89
	Tertiary	1657	24.22

Regarding sector of residence, the study shows that approximately 61% of the respondent are living in the urban sector. The probable reason may not be disconnected from the fact that the study was conducted via phone survey which may be more realistic to carry out in the urban area compare to the rural area which may have difficulty on mobile network provision. More than 70% of the respondents were married while 10.79% are single. Meanwhile, 57.63% of the households have a size of members between 6 and 10 household members. The average household size is 6.47 with a standard deviation of 3.79. Regarding education of the respondents, 35.26% of the respondent obtained primary education while 24.22% has obtained tertiary education, these statistics are I consonance with existing literature from Nigeria [84–86].

### Shocks experienced by the households due to COVID-19 pandemic

**Fig. 2:**
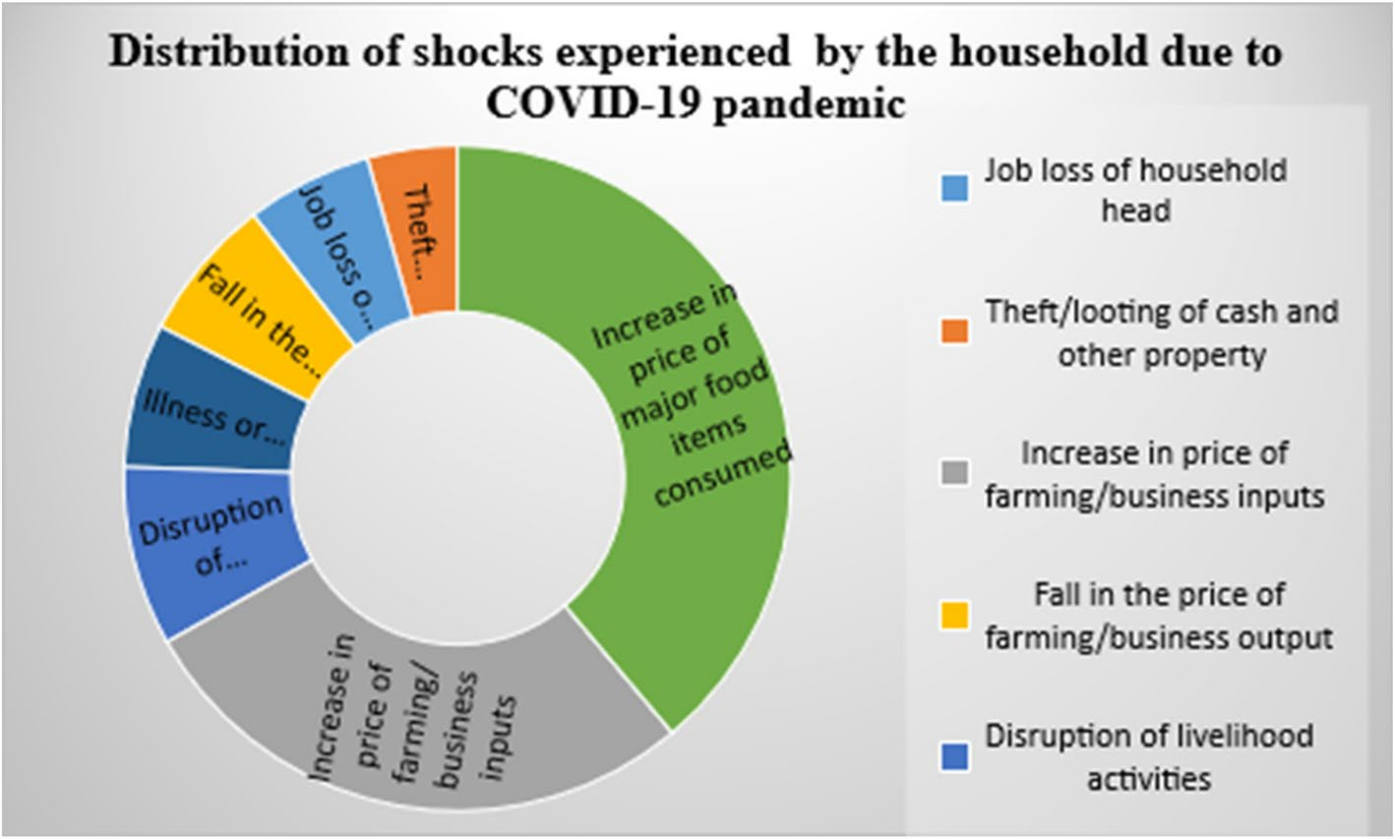
Distribution of shocks experienced by the household due to COVID-19 pandemic

The result shows that 94% of the household’s experience at least one of the COVID-19 induced shocks. The results suggest that the respondents are 9 out of 10 of the respondents were affected by the COVID-19 pandemic. As shown in Fig. 2, the highest (89%) shock experienced was associated to increase food prices followed by increase in price of farming/business inputs (64%). The average COVID-19 induced shocks experienced by the households is 2.69, indicating each household experience approximately 3, COVID-19 induced shocks (Table 1), this conforms with the findings of [87].

### Measurement of output variables

**Fig. 3:**
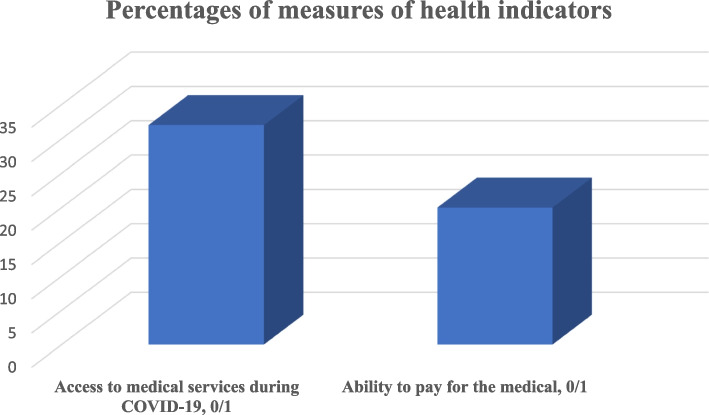
Percentages of measures of health indicators

The focus of measuring human capital development on this study focused on education and health outcomes. Firstly, we measure the health outcomes using two variables (1) Access to medical services by the household members (2) Ability to pay for the medical services by the household. The result shows that 32% of the households had access to medical services during COVID-19 (the trend from different rounds of survey will detailed later) while 20% were able to afford. The Fig. 3 depicts that the challenge is more of affordability rather than accessibility.

### Gender distribution of percentage to the health indicators

**Fig. 4:**
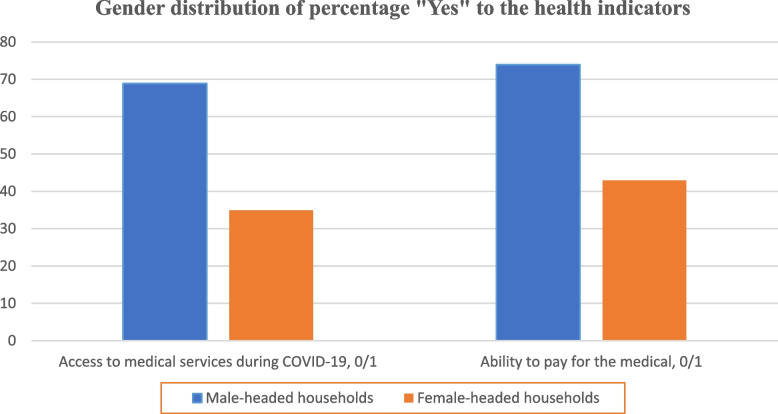
Gender distribution of percentage “Yes” to the health indicators

We present in Fig. 4 the gender distribution of respondents based on the health indicators. For both two indicators, the result shows that the male-headed households were able to access and afford medical services compared to female-headed counterparts. Studies has shown that accessing basic amenities, male-headed households have a higher probability of accessing good medical health compared to female counterparts [88–90].

### Percentages of measures of education indicators

**Fig. 5:**
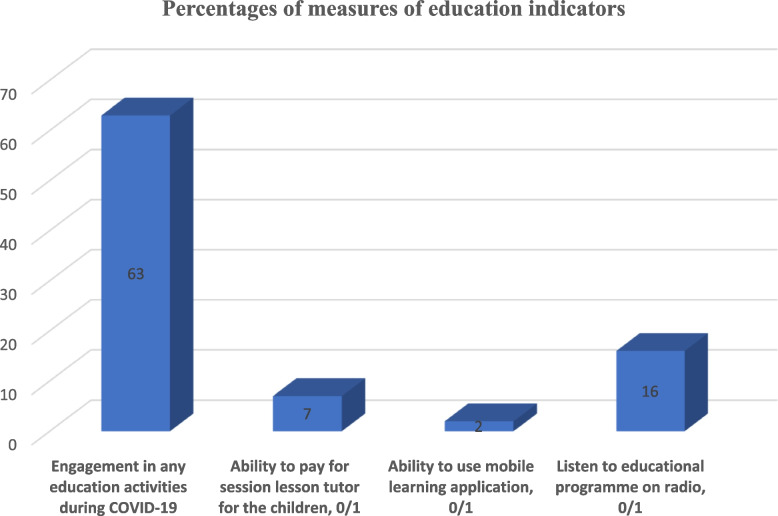
Percentages of measures of education indicators

Secondly, we measured the education outcomes using four variables – (1) Engagement in any education activities during COVID-19 (2) Ability to use mobile learning application (3) Ability to pay for session lesson tutor for the children (4) Listen to educational programme on radio by the children. Regarding the educational outcome, 63% of the children in the household were able to had educational engagement but only 7% were able to afford to pay for home tutor. The use of mobile learning application is significantly low among the respondents which perhaps may suggest the level of economic status of the households [91]. Figure 5 shows that just 2% of the respondents has children that use mobile learning application in Nigeria during the COVID-19 pandemics.

### Gender distribution of percentage “yes” to the education indicators

**Fig. 6:**
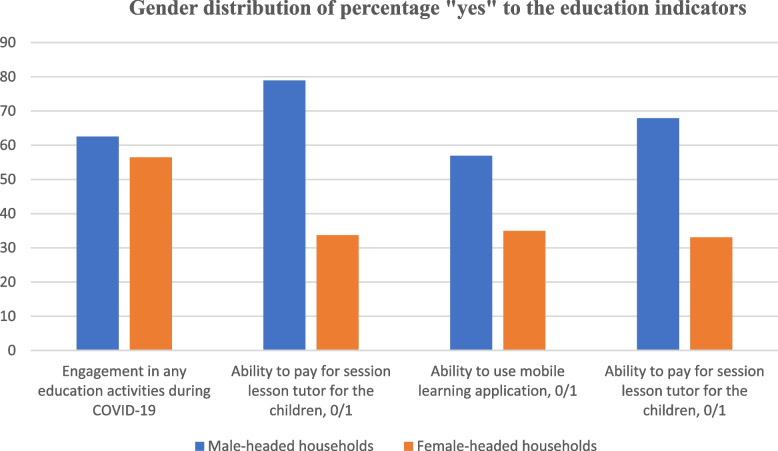
Gender distribution of percentage “yes” to the education indicators

Furthermore, Fig. 6 depicts the gender distribution of respondents based on the education indicators. The outcomes were disaggregated on gender basis using four variables – (1) Engagement in any education activities during COVID-19 (2) Ability to use mobile learning application (3) Ability to pay for session lesson tutor for the children (4) Listen to educational programme on radio by the children. Across the four indicators, the result shows that the households headed by males were able to access and afford educational services compared to female-headed counterparts. This conforms with previous studies that accessing basic amenities such as education, male-headed households have a higher probability of accessing good educational facilities compared to their female counterparts [92–95].

### Health outcomes of the participants

Equations (1) and (2) shows how health outcomes have varied with the shocks induced by COVID-19 pandemic. Table 3 shows the implications of the shocks induced by the spread of the pandemic on the households’ health outcomes, measured as binary indicators of access to medical services, and ability to pay for medical services. The overall health outcomes were also measured by reporting positive in the two binary outcomes. The number of shocks experienced by households due to COVID-19 pandemic for each household are transformed using an inverse hyperbolic sine transformation, to accommodate those households with zero reported shocks.^1^

**Table 3:** Effect of COVID-19 induced shocks on health outcomes: Dummy (At least one shock)

Variables	(1) Access to medical services	(2) Ability to pay for medical services	(3) Health
Gender (1 = male)	0.685^***^	0.270	0.227^***^
	(0.158)	(0.206)	(0.0812)
Age	-0.001	-0.002	-0.003
	(0.003)	(0.005)	(0.001)
Household size	0.0974^***^	0.00751	0.0690^***^
	(0.0191)	(0.0210)	(0.00825)
Primary occupation (1 = agriculture)	-0.0950	-0.123	-0.124^*^
	(0.142)	(0.173)	(0.0653)
Shock (dummy)	-0.175	-0.110^***^	-0.188^**^
	(0.294)	(0.017)	(0.114)
Social safety nets [SSN] (1 = yes)	0.0529^***^	0.152^***^	0.542^***^
	(0.00398)	(0.0022)	(0.080)
Shock ^*^ SSN	0.111^***^	0.202^***^	0.211^***^
	(0.005)	(0.001)	(0.009)
Other controls	YES	YES	YES
Round FE	YES	YES	YES
Observations	6,840	6,840	6,840
Number of round	4	4	4

Standard errors in parentheses

^***^p < 0.01

^**^p < 0.05

^*^p < 0.1

The interaction between shocks and access to social safety nets dummy captures the mediating effects in the evolution of our outcomes of interest associated with varying exposure to the spread of the pandemic. A negative and significant coefficient shows that households registering higher numbers of shocks are likely to experience greater decrease in the probability of positive health outcomes. The coefficients in Table 3 show that experiencing at least one of the shocks induced by COVID-19 cases is associated with probability of households’ inability to pay for medical services. Correspondingly, experiencing at least one of the shocks induced by COVID-19 pandemic is associated with a significant decrease in the aggregate health outcomes indicator.

The results show that the shocks reduce the probability of having good health outcome by 28.8%. More so, we mediated the role of access to social safety nets (SSN) by interacting the dummy variable of the shocks with access to SSN. The results show a positive and significant association with the health indictors. The result suggests that access to social safety nets plays a cushioning effect of the negative effect of shocks induced by COVID-19 pandemic, this corroborates similar studies in the developing nations [23, 87, 97–99].

We further report in Table 4 the results of the association of the intensity of the shocks induced by COVID-19 pandemic and health outcomes using the numbers of shock experienced. To facilitate this, we counted the number of positive response to shock i.e. households assuming a value of 1 if experience a shock. A negative and significant coefficient shows that households registering higher numbers of shocks are likely to experience greater decrease in the probability of good health outcomes. Unlike the dummy estimation, the number of shocks is negatively and significantly associated with all the two health indicators and subsequently health outcomes. The result suggests a higher association even in term of the coefficients. For instance, Table 4 shows that intensity of shocks induced by COVID-19 pandemic is negatively and significantly associated with access to medical services and ability to pay for medical bills, suggesting the doubling or increase in the number of shocks induced by COVID-19 pandemic reduce probability of health outcomes.

**Table 4:** Effect of COVID-19 induced shocks on health outcomes: Extent (Number of shocks)

Variables	(3)	(4)	(5)
	Access to medical services	Ability to pay for medical services	Health
Gender (1 = male)	0.706^***^	0.361^*^	0.176^**^
	(0.159)	(0.210)	(0.0818)
Age	-0.000763	-0.00459	-0.000949
	(0.00398)	(0.00523)	(0.00200)
Household size	-0.0995^***^	0.0302	-0.0606^***^
	(0.0193)	(0.0222)	(0.00831)
Primary occupation (1 = agriculture)	-0.107	-0.104	0.0872
	(0.142)	(0.174)	(0.0659)
Shock (count)	-0.338^***^	-0.544^***^	-0.771^***^
	(0.0047)	(0.0421)	(0.0180)
Social safety nets (1 = yes)	0.0885^***^	0.053^***^	0.055^***^
	(0.0103)	(0.0122)	(0.0001)
Shock ^*^ SSN	0.256^*^	0.133^*^	0.201^*^
	(0.145)	(0.800)	(0.116)
Other controls	YES	YES	YES
Round FE	YES	YES	YES
Observations	6,840	6,840	6,840
Number of round	4	4	4

Standard errors in parentheses

^***^p < 0.01

^**^p < 0.05

^*^p < 0.1

Similarly, the result suggests that increase in the number of shocks induced by COVID-19 pandemic is associated with a significant decrease in the aggregate health outcomes indicator. The result is consistent with the finding of [100, 101] suggesting that shocks (such as the one induced by COVID-19 pandemic) reduced access to healthcare services when household members are confronted with an illness. To jointly examine the meditating effects of COVID-19 induced shocks and access to social safety nets, we interact the variables to estimate the association with the health outcomes. The result suggests a weaker significant but positive association of the interacted variable with the health outcomes.

The weaker significant association may be pointing to the fact that as the intensity of shocks is increasing, just an access to a social safety nets may not be sufficient to cushion the negative effects induced by the pandemic rather the depth and value of the social safety nets may be appropriate to match the intensity of the shocks. Collectively, our findings suggested, similar to the study of [102] that effectively boosting household resilience may require significant transfers perhaps over multiple years. Hence, just an access to the national social safety nets programs or social programs that contribute or transfer insignificant amounts to beneficiaries over limited time horizons may not be effective as expected.

### Education outcomes of the respondents

Reduction in household income and purchasing capacity is one of the most significant pathways through which the COVID-19 pandemic can affect household non-food expenditure [87, 103, 104]. Results in Table 5 and 6 shows the implication of the spread of the pandemic through the shocks induced on education outcomes. The education outcomes were captures using 4 indicators (1) Engagement in any education activities during COVID-19 (2) Ability to use mobile learning application (3) Ability to pay for session lesson tutor for the children and (4) Listen to educational programme on radio by the children.

**Table 5:** Effect of COVID-19 induced shocks on education outcomes: Dummy (At least a shock)

Variables	(1)	(2)	(3)	(4)	(5)
	Engage	Tutor	Mobile	Radio	Education
Gender (1 = male)	0.273^***^	0.277^*^	-0.0284	0.133	0.216^***^
	(0.0977)	(0.157)	(0.192)	(0.147)	(0.0810)
Age	-0.0002	0.001	0.005	0.004	-0.002
	(0.00254)	(0.00386)	(0.00489)	(0.00369)	(0.00198)
Household size	-0.041^***^	-0.0154	0.0182	0.00784	0.117^***^
	(0.00946)	(0.0147)	(0.0174)	(0.0136)	(0.00883)
Primary occupation (1 = agriculture)	-0.378^***^	0.0204	-0.173	-0.450^***^	-0.181^***^
	(0.0758)	(0.111)	(0.141)	(0.104)	(0.0651)
Shock (dummy)	-0.402^***^	-0.573^**^	-0.302^***^	-0.181	-0.394^***^
	(0.132)	(0.247)	(0.005)	(0.554)	(0.115)
Social safety nets (1 = yes)	0.1124^***^	0.2204^***^	0.0411^***^	0.1874^***^	0.585^***^
	(0.002)	(0.001)	(0.0112)	(0.022)	(0.0768)
Shock ^*^ SSN	0.100^***^	0.143^***^	0.271^***^	0.223^***^	0.459^***^
	(0.005)	(0.001)	(0.014)	(0.021)	(0.022)
Other controls	YES	YES	YES	YES	YES
Round FE	YES	YES	YES	YES	YES
Observations	6,840	6,840	6,840	6,840	6,840
Number of round	4	4	4	4	4

Standard errors in parentheses

^***^p < 0.01

^**^p < 0.05

^*^p < 0.1

**Table 6:** Effect of COVID-19 induced shocks on education outcomes: Extent (Number of shocks)

Variables	(1)	(2)	(3)	(4)	(5)
	Engage	Tutor	Mobile	Radio	Education
Gender (1 = male)	0.254^***^	0.207	-0.0399	0.0573	0.198^**^
	(0.0979)	(0.158)	(0.193)	(0.149)	(0.0811)
Age	-0.000358	0.00237	0.00588	0.00534	-0.00275
	(0.00254)	(0.00387)	(0.00489)	(0.00369)	(0.00198)
Household size	-0.0428^***^	-0.0283*	0.0170	-0.00478	0.115^***^
	(0.00950)	(0.0152)	(0.0175)	(0.0140)	(0.00888)
Primary occupation (1 = agriculture)	-0.389^***^	-0.0564	-0.186	-0.377^***^	-0.190^***^
Shock (count)	-0.485**	-0.186^***^	-0.259^***^	-0.197^***^	-0.1544^***^
	(0.0202)	(0.0306)	(0.0399)	(0.0292)	(0.0177)
	(0.0763)	(0.112)	(0.143)	(0.105)	(0.0652)
Social safety nets (1 = yes)	0.300^***^	0.220^***^	0.133^***^	0.322^***^	0.589^***^
	(0.0225)	(0.0115)	(0.021)	(0.025)	(0.0797)
Shock * SSN	0.330	0.444	0.599	0.223^***^	0.859
	(0.322)	(0.322)	(0.711)	(0.001)	(0.810)
Other controls	YES	YES	YES	YES	YES
Round FE	YES	YES	YES	YES	YES
Observations	6,840	6,840	6,840	6,840	6,840
Number of round	4	4	4	4	4

Standard errors in parentheses

^***^p < 0.01

^**^p < 0.05

^*^p < 0.1

As expected, the spread of the COVID-19 pandemic through the induced shocks is associated with a significant reduction in education indicators and aggregated education outcomes [105, 106]. The results show that all the indicators, except the coefficient of listening to educational programme on radio by the children, were significant and negatively associated with health outcomes. The result shows a higher coefficient for the association of shocks on ability of household to pay for session lesson tutor for the children. The result suggests that the children in the household will have reduced probability of having a session lesson with a tutor for at least 57.3% and 40.2% for engagement in any education activities if the household experience at least one of the shocks induced by COVID-19 pandemic. Similarly, the shocks shown a negative association with aggregate education outcomes. The results suggest that, overall, household that experience at least one of the shock will less likely to have adequate education outcomes compare to household without any of the shocks which corroborates existing literature [6, 95, 107, 108].

In most cases, the primary objective of social safety nets programs is to improve poor households’ resilience through addressing food and non-food insecurity while reducing vulnerability to various types of shocks [87, 109]. Expectedly, the access to social safety nets indicate a positive and consistent association with the education outcomes. The estimate shown in Table 5 consistently shows that access to social safety net improve education outcomes in all the indicators [95]. The results suggest that those children in households who receive social support experience a significant increase in the likelihood of engagement in any education activities during COVID-19 and ability to use mobile learning application. In addition, the result suggests that social safety nets may likely cushion effect associated with COVID-19 pandemic by increasing the probability of the households to be able to pay for session lesson tutor for the children and also listen to educational programme on radio by the children.

In addition, the interaction of the two dummy variables of interest—experience of any shocks and access to social safety—were found to be positively associated with education outcomes despite the spread of the pandemic and associated lockdowns. The results show that despite the shock induced by the pandemic, the probability to pay for the service of a tutor increase by 14.3% suggesting a moderating effect of access to the social safety nets. The likelihood to engage in any education activities during the pandemic for the children despite the shock induced by the pandemic increase by 10%. The result suggests consistent association of the interacted variables and the education outcome.

In the same vein, the health outcomes reported in Table 6 shows the results of the association of the intensity of the shocks induced by COVID-19 pandemic and education outcomes using the numbers of shock experienced. Similar method was used to facilitate this, we counted the number of positive response to shock i.e. households assuming a value of 1 if experience a shock. Correspondingly, there is negative associations in all the indicators of education outcomes and positive association for the access to safety nets as shown in Table 6.

However, there is a twist on the association of the education outcomes and the interaction of the intensity of shocks induced by COVID-19 pandemic and access to social safety nets. Except for listening to educational programme on radio by the children, none of the education indicators were significantly associated with the interaction between access to social safety nets and intensity of shocks induced by COVID-19 pandemic. Although the coefficients were positive but they were insignificant. The probable reason for this can be explained in the context of expenditure prioritization. In the face of competition for the limited household resources, food related expenditure may take higher priority than non-food expenditure.

Meanwhile, for non-food expenditure, health related expenditure may take higher priority than education [110, 111]. Hence, with many conflicting demands for household resources, the effect of the social safety nets may not be adequate if the depth and value of the social support to mitigate the huge negative impacts by the by COVID-19 pandemic. This further intensify the points on the mediating role of social safety nets. This further reiterates the consistency and value of emergency social safety nets to cushion the negative impacts of shocks such as COVID-19 pandemic.

## Conclusion and policy recommendation

We utilised up-to-date nationally representative data from Nigeria to examine the impact of COVID-19 produced shocks on the development of families’ human capital, using health and education outcomes as indicators. We examined the extent to which access to social safety nets helps mitigate the adverse impacts of shocks caused by the COVID-19 pandemic. Our analysis indicates that the disruptions caused by the rapid spread of the pandemic had substantial impacts on the health and education outcomes reported by the households in our sample. Notably, having access to social safety nets was found to have a beneficial correlation with health and education outcomes. The outcomes of the interplay between access to social safety nets and shocks generated by the COVID-19 pandemic were inconclusive when considering a dummy variable to measure the shocks, as well as when accounting for the amount of shocks experienced. Nevertheless, when it comes to health outcomes, the combined variables were discovered to have a favourable albeit feeble correlation. The findings indicate that while access to social safety nets can help alleviate the adverse impacts of COVID-19 shocks, it is important to ensure the long-term viability of this mediating function. Regarding the education result, only one of the four education indicators showed significant interaction variables, specifically the children’s engagement in listening to educational programmes on the radio. Nevertheless, the impact of social safety nets on the overall education outcome was determined to be beneficial, albeit not statistically significant. Our findings indicate that effectively enhancing the ability of households to withstand and recover from shocks, as well as encouraging the development of human skills, may necessitate substantial and innovative measures that could span several years. Therefore, mere access to national social safety nets programmes or social programmes that provide little sums of assistance to recipients for a short period of time may not provide the desired results.

## Data Availability

The data used in this study is not publicly available due to the confidential policy but are available from the corresponding author on reasonable request.

## Footnotes

1 As we have large positive values of shocks for most households, such a transformation is expected to be innocuous (e.g., [96]). 
